# Retinal Organoid-Derived Exosomes Reduce CNV Lesion and Restore RPE Integrity in Mouse Laser-Induced Choroidal Neovascularization (CNV) Model

**DOI:** 10.3390/ijms262311327

**Published:** 2025-11-24

**Authors:** Jin Young Yang, Yeji Kim, Sumin An, Jung Woo Han, Jun-Sub Choi, Tae Kwann Park

**Affiliations:** 1 Laboratory for Translational Research on Retinal and Macular Degeneration, Soonchunhyang University Bucheon Hospital, Bucheon 14584, Republic of Korea; yjy841012@sch.ac.kr (J.Y.Y.); yeiji77@naver.com (Y.K.); sue07104@gmail.com (S.A.); 106236@schmc.ac.kr (J.W.H.); mjschoi69@gmail.com (J.-S.C.); 2 Department of Ophthalmology, Soonchunhyang University Bucheon Hospital, Bucheon 14584, Republic of Korea; 3 Department of Interdisciplinary Program in Biomedical Science, Soonchunhyang Graduate School, Soonchunhyang University Bucheon Hospital, Bucheon 14584, Republic of Korea

**Keywords:** neovascular age-related macular degeneration, choroidal neovascularization, exosome, retinal organoids, human induced pluripotent stem cell, mitogen-activated protein kinase

## Abstract

To address the shortcomings of existing anti-VEGF monotherapy in neovascular age-related macular degeneration (nAMD), we investigated the therapeutic capabilities of exosomes obtained from human induced pluripotent stem cell (hiPSC)-derived retinal organoids in a mouse model of laser-induced choroidal neovascularization (CNV). To evaluate Retinal Organoid-derived exosome (RO-Exo) distribution after intravitreal (IVT) injection, calcein-labeled RO-Exo was observed using confocal microscopy. CNV was induced in C57BL/6 J mice by laser photocoagulation. RO-Exo was isolated from retinal organoids (differentiation days 55–65) and injected 5 days post-laser. Therapeutic efficacy was evaluated on day 12. Vascular leakage and CNV size were assessed by angiography and CD31 immunostaining. We also examined HIF-1α/VEGF-A expression (Western blotting), Retinal Pigment Epithelium (RPE) integrity markers (immunofluorescence staining for α-SMA, fibronectin, and ZO-1), and the activation of the Mitogen-Activated Protein Kinase (MAPK) pathway (phospho-ERK, -p38, -JNK) in CNV lesions. After IVT injection, RO-Exo migrated to the RPE layer, showing high retinotropic distribution. In the CNV model, RO-Exo significantly reduced vascular leakage and CNV size, with greater suppression of HIF-1α and VEGFA expression than aflibercept, the standard-of-care anti-VEGF drug. CD31-positive vasculature was decreased, accompanied by downregulation of fibronectin (a fibrotic marker) and restoration of RPE hexagonality and integrity. Furthermore, RO-Exo inhibited the activation of ERK, P38, and JNK in CNV lesions. Our study results demonstrate that RO-Exo exhibits multi-target therapeutic effects—including anti-angiogenic, anti-fibrotic, and neuroprotective actions—offering a promising alternative to conventional anti-VEGF therapy for nAMD.

## 1. Introduction

Age-related macular degeneration (AMD) is a leading cause of irreversible blindness and severe visual impairment among individuals aged 65 years and older, particularly in Western populations [1,2,3]. AMD is classified into two forms: dry (atrophic or geographic atrophy) and wet (neovascular). The choroidal neovascularization (CNV) in the macular region is a characteristic feature in wet AMD. The new vessels grow from the choroid, breach Bruch’s membrane and invade the subretinal spaces. This macular neovascularization (MNV) induces a disruption of retinal structure and progressive central vision loss [4,5,6,7]. Among its clinical subtypes, neovascular AMD (nAMD) is characterized by the pathological proliferation of CNV. Although anti-vascular endothelial growth factor (anti-VEGF) agents are widely utilized as standard therapeutic interventions, their efficacy in mitigating subretinal fibrotic remodeling and retinal neurodegeneration remains suboptimal. This limitation highlights the imperative for novel therapeutic strategies capable of concurrently modulating multiple pathogenic mechanisms. Currently, the standard treatment for wet AMD is intravitreal injection of anti-VEGF drugs (Ranibizumab, Aflibercept and Brolucizumab) [3,8]. Although these treatments effectively inhibit neovascular growth and reduce vascular leakage, their therapeutic efficacy is limited by frequent administration, poor long-term compliance, and insufficient therapeutic effects in some patient groups [9,10]. Furthermore, anti-VEGF monotherapy does not address subretinal fibrosis and chronic inflammation, which are closely related to irreversible visual loss. Long-term VEGF blockade can disrupt neuroretinal homeostasis by interfering with physiological processes in the retina [11].

Exosomes are nano-sized extracellular vesicles (typically 30–150 nm) secreted by nearly all cell types. They originate from multivesicular bodies within cells and are released into the extracellular space, carrying a rich cargo of proteins, lipids, DNA and various RNA types (including mRNA, miRNA, and lncRNA). These vesicles act as messengers, facilitating intercellular communication and influencing the behavior of recipient cells [12,13,14,15,16,17]. Over recent years, these vesicles have attracted significant attention across biomedical disciplines—including cancer research and neurological diseases—owing to their low immunogenicity, intrinsic biocompatibility, and ability to traverse physiological barriers. In ophthalmology, a growing body of evidence highlights the therapeutic potential of exosomes, particularly those derived from stem cells. These stem cell-derived exosomes have demonstrated remarkable anti-inflammatory and anti-angiogenic effects in models of retinal degeneration, corneal injury, and glaucomatous neuropathy. Their ability to modulate immune responses, promote neuroprotection, and deliver therapeutic molecules suggests a novel, cell-free approach to managing ocular diseases. As exosome-based strategies continue to evolve, they offer new hope for targeted intervention and regeneration in otherwise treatment-resistant eye conditions [18,19,20]. However, these exosomes lack retinal specificity and may not fully recapitulate the complex signaling environment of the developing retina. Their effects on fibrotic remodeling and neuroprotection under CNV-related stress also remain insufficiently defined.

Retinal organoids (RO) derived from human induced pluripotent stem cells (hiPSCs) mimic the layered architecture and cellular diversity of the human retina, including photoreceptors and retinal ganglion cells [21,22]. During early stages of retinal differentiation, these organoids exhibit stage-specific expression of key transcription factors involved in retinal neurogenesis, laminar organization, and cell fate specification, reflecting developmental signaling cascades critical for retinal tissue formation [23]. The paracrine factors secreted by these developmentally relevant organoids are hypothesized to provide a biologically specialized and complex signaling cargo within RO-Exo, suggesting their potential to address multiple aspects of AMD pathology concurrently [24]. In our previous work, we demonstrated that exosomes derived from early-stage retinal organoids (RO-Exo) conferred photoreceptor protection and attenuated retinal degeneration by suppressing mitogen-activated protein kinase (MAPK) signaling in a Royal College of Surgeons (RCS) rat model [25].

In this study, we aimed to evaluate the therapeutic efficacy of RO-Exo in a laser-induced CNV mouse model, demonstrating its ability to suppress neovascularization and fibrosis, while simultaneously preventing retinal neuronal loss and preserving retinal pigment epithelial (RPE) characteristics. These results suggest that RO-Exo, with its retinal-specific origin and highly biocompatible molecular composition, has potential therapeutic effects on neovascular AMD and can be utilized as a multi-targeted therapeutic approach, particularly in cases that do not respond to anti-VEGF monotherapy.

## 2. Results and Discussion

### 2.1. Retinal Organoids Differentiation and Exosomes Isolation

**Figure 1:**
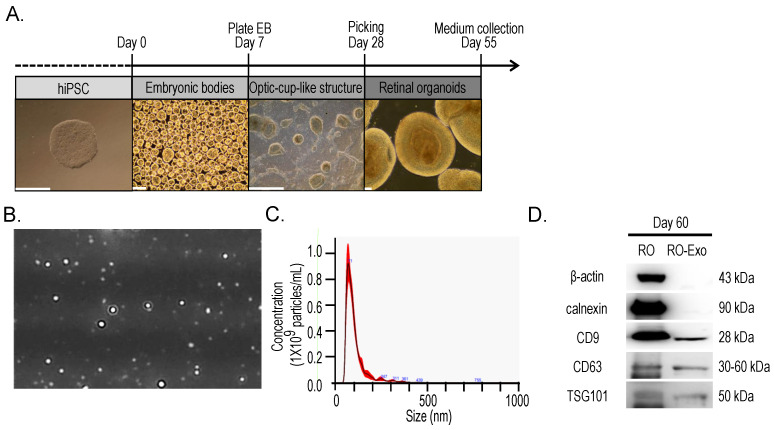
Differentiation of retinal organoids from hiPSCs and isolation of retinal organoid–derived exosomes (RO-Exo). (**A**) Schematic of the stepwise differentiation process of human induced pluripotent stem cells (hiPSCs) into retinal organoids, including embryoid body (EB) formation, optic cup-like structure development, and maturation into retinal organoids by day 60. Representative brightfield images are shown for each stage. Scale bars = 500 µm. (**B**) Representative nanoparticle tracking analysis (NTA) image of RO-derived exosomes. (**C**) NTA profile showing a mode size of 73.50 ± 3.60 nm and a concentration of 1.10 × 10^9^ particles/mL. (**D**) Western blot analysis confirming successful isolation of RO-derived exosomes. Exosomal markers (CD9, CD63, TSG101) were detected in both RO and RO-Exo, while cellular markers (β-actin, calnexin) were only observed in RO lysates.

To obtain exosomes from retinal organoids (RO-Exo), human induced pluripotent stem cells (hiPSC) were differentiated into retinal organoids using a stepwise protocol. This differentiation process included the formation of embryoid bodies (EB), the development of optic cup-like structures, and the maturation into retinal organoids by day 55 (Figure 1A). Conditioned medium was collected from mature organoids between days 55 and 65, and exosomes were subsequently isolated from this medium. Nanoparticle tracking analysis (NTA) showed that the average exosome size was 71.50 ± 3.60 nm at a concentration of 1.10 × 10^9^ particles/mL (Figure 1B,C). Western blot analysis of protein lysates from RO and RO-derived exosomes (RO-Exo) at day 60 showed the presence of exosomal markers CD9, CD63, and TSG101 in both samples. In contrast, the cytosolic marker β-actin and the endoplasmic reticulum marker calnexin were identified only in RO lysates, suggesting that the RO-Exo preparation was devoid of cellular and organelle contamination (Figure 1D).

### 2.2. Retinal and RPE Distribution of Calcein-Labeled RO-Exo in Mice

**Figure 2:**
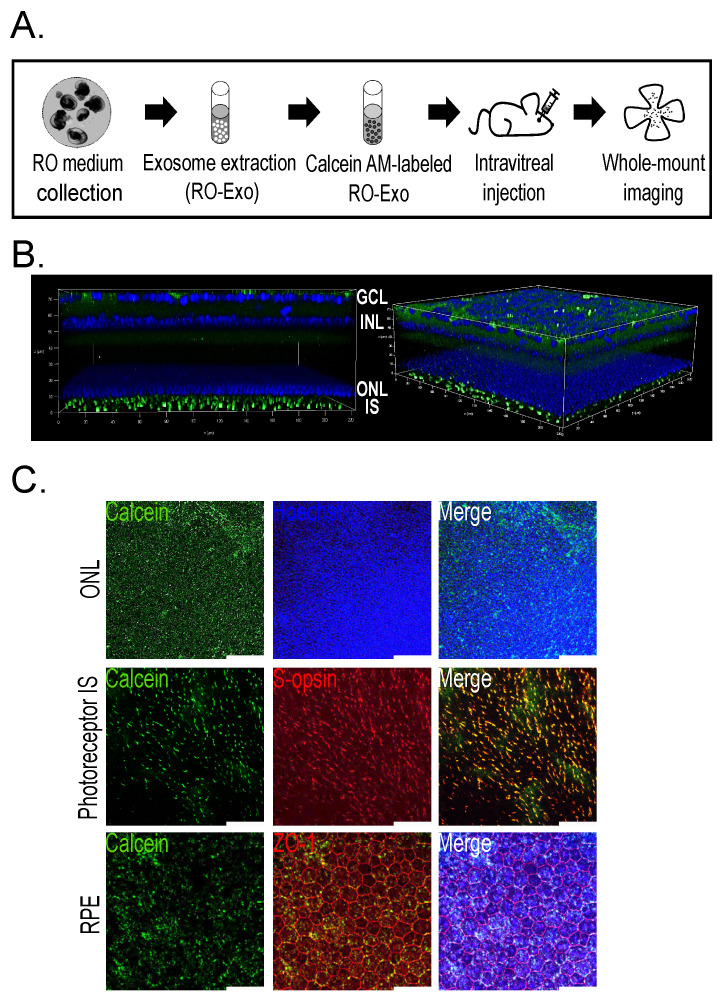
Tropism of Calcein-labeled RO-Exo in mouse retinal and RPE cells. (**A**) Schematic showing the experimental design with intravitreal injection (IVT) of Calcein-labeled RO-Exo followed by retinal imaging performed 3 h after injection. (**B**) Z-stack confocal image showing Calcein signals in the retinal layer (green). (**C**) Single-plane confocal images demonstrating intracellular uptake of RO-Exo, with co-localization with nuclei in the ONL (top), presence in S-opsin (+, red) cone inner segments (middle), and localization in both cytoplasm and nuclei of ZO-1 (+, red) RPE cells (bottom). Scale bars = 50 µm.

To assess the in vivo distribution of RO-Exo, exosomes were labeled with Calcein-AM, a membrane-permeable dye that becomes fluorescent upon esterase-mediated hydrolysis. As depicted in the experimental workflow figure, 1 μL of Calcein-labeled RO-Exo was administered via intravitreal injection into C57BL/6 J mice. Ocular tissues were collected 3 h post-injection and subjected to whole-mount confocal microscopy (Figure 2A).

Z-stack confocal imaging revealed Calcein-positive signals throughout the ganglion cell layer (GCL), inner nuclear layer (INL), outer nuclear layer (ONL), and photoreceptor inner segments (IS), indicating broad distribution of RO-Exo across multiple retinal layers (Figure 2B). High-resolution imaging of the ONL demonstrated Calcein fluorescence localized near Hoechst-stained nuclei, suggesting uptake by photoreceptor cell bodies (Figure 2C, top). Moreover, Calcein signals colocalized with S-opsin–positive inner segments of cone photoreceptors (Figure 2C, middle), supporting internalization by cone cells. In the retinal pigment epithelium (RPE), Calcein fluorescence was detected in both the cytoplasm and nuclei of ZO-1–outlined cells, confirming efficient intracellular delivery of RO-Exo to RPE cells (Figure 2C, bottom). These findings demonstrate rapid and efficient internalization and widespread retinal and RPE distribution of RO-Exo within 3 h of administration, supporting their utility for efficient in vivo therapeutic delivery. Calcein-positive signals were detected in retinal and RPE cells as early as 3 h post-injection, indicating rapid uptake and distribution of RO-Exo across multiple ocular layers.

### 2.3. Vascular Leakage Suppression by RO-Exo

**Figure 3:**
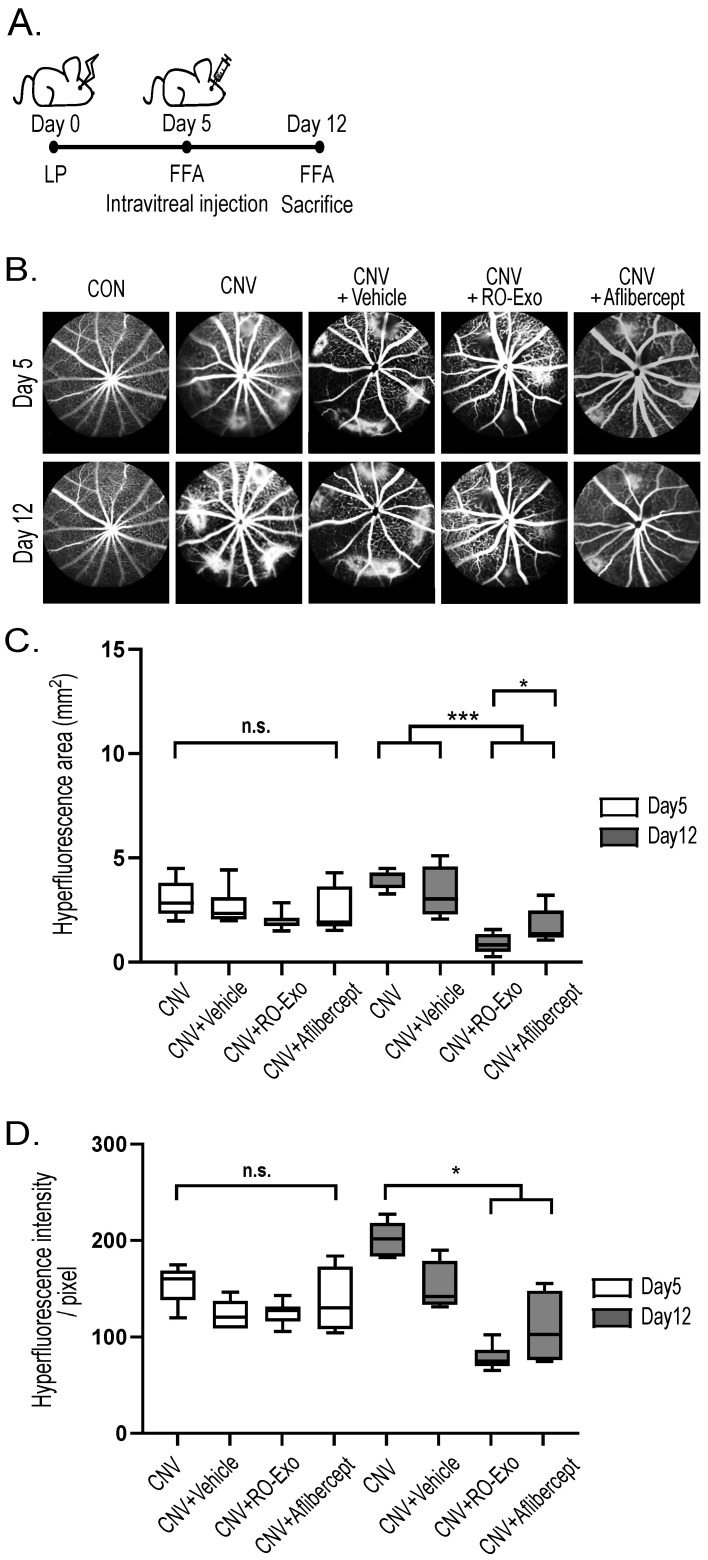
RO-Exo reduces vascular leakage in laser-induced CNV. (**A**) Experimental timeline showing laser photocoagulation (LP) performed on day 0, intravitreal injection (IVT) on day 5, and fundus fluorescein angiography (FFA) conducted on days 5 and 12. (**B**) Representative FFA images of each group at days 5 and 12. Scale bar = 200 µm. (**C**) Quantification of hyperfluorescent lesion area (mm^2^). n.s. indicates not significant. (**D**) Quantification of hyperfluorescent intensity (pixel). n.s. indicates not significant. Bar graph data are presented as the mean ± standard error of the mean (S.E.M.). Statistical significance was denoted as *p* < 0.05 (*) and *p* < 0.001 (***) using the Mann–Whitney U test.

As illustrated in the experimental timeline (Figure 3A), CNV lesions were confirmed by fundus fluorescein angiography (FFA) on day 5 post-laser photocoagulation (LP), followed by intravitreal injection of RO-Exo, vehicle, or aflibercept. On day 12 post-LP, FFA imaging (Figure 3B) was performed to evaluate vascular leakage. FFA is a diagnostic and evaluation technique for retinal neovascularization, where in highly leaky vessels, the injected fluorescein dye escapes outside the vasculature, forming hyperfluorescence. The size and intensity of this hyperfluorescence directly indicate the degree of vascular leakage. Quantitative analysis revealed that both the RO-Exo–treated and aflibercept-treated groups exhibited significantly reduced hyperfluorescent area compared to the CNV groups (w/wo vehicle) (*p* < 0.001; Figure 3C). The RO-Exo–treated group showed a significantly greater reduction than the aflibercept-treated group (*p* < 0.05), suggesting superior efficacy. The hyper-fluorescence intensity was relatively lower in the RO-Exo–treated group compared to the aflibercept-treated group (Figure 3D). These findings demonstrate that RO-Exo attenuates CNV-associated vascular leakage and support its potential as a therapeutic approach for neovascular retinal diseases.

### 2.4. Reduction in HIF-1α and VEGFA Expression by RO-Exo

**Figure 4:**
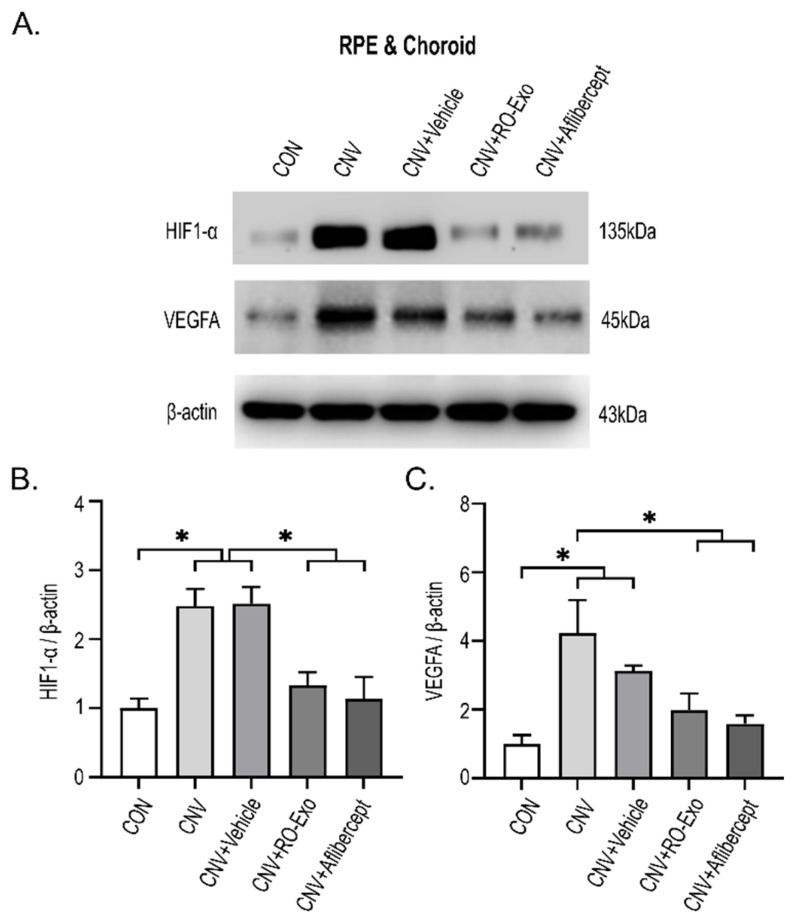
RO-Exo suppresses angiogenesis-related protein expression in the RPE–choroid complex. (**A**) Western blot images of HIF-1α and VEGFA in the RPE–choroid complex on day 12 post-LP. (**B**,**C**) Quantification of HIF-1α (**B**) and VEGFA (**C**) expression levels normalized to β-actin. Bar graph data are presented as the mean ± standard error of the mean (S.E.M.). Statistical significance was denoted as *p* < 0.05 (*), using the Mann–Whitney U test.

To investigate whether RO-Exo modulates hypoxia-driven angiogenic signaling, we analyzed the expression levels of hypoxia-inducible factor 1-alpha (HIF-1α) and vascular endothelial growth factor A (VEGFA) in the RPE–choroid complex. Both proteins were significantly upregulated in the CNV and CNV + vehicle groups compared to the control group (*p* < 0.05), reflecting hypoxia-induced proangiogenic responses. RO-Exo treatment significantly reduced HIF-1α expression compared to both CNV groups (w/wo vehicle) (*p* < 0.05). VEGFA expression was also significantly decreased compared to the CNV group (*p* < 0.05). No significant difference was observed between the RO-Exo and aflibercept groups, indicating comparable levels of suppression (Figure 4A–C). RO-Exo attenuated CNV-associated angiogenic signaling by reducing HIF-1α and VEGFA expression, with effects comparable to aflibercept.

### 2.5. Suppression of Pathologic Neovascularization by RO-Exo

**Figure 5:**
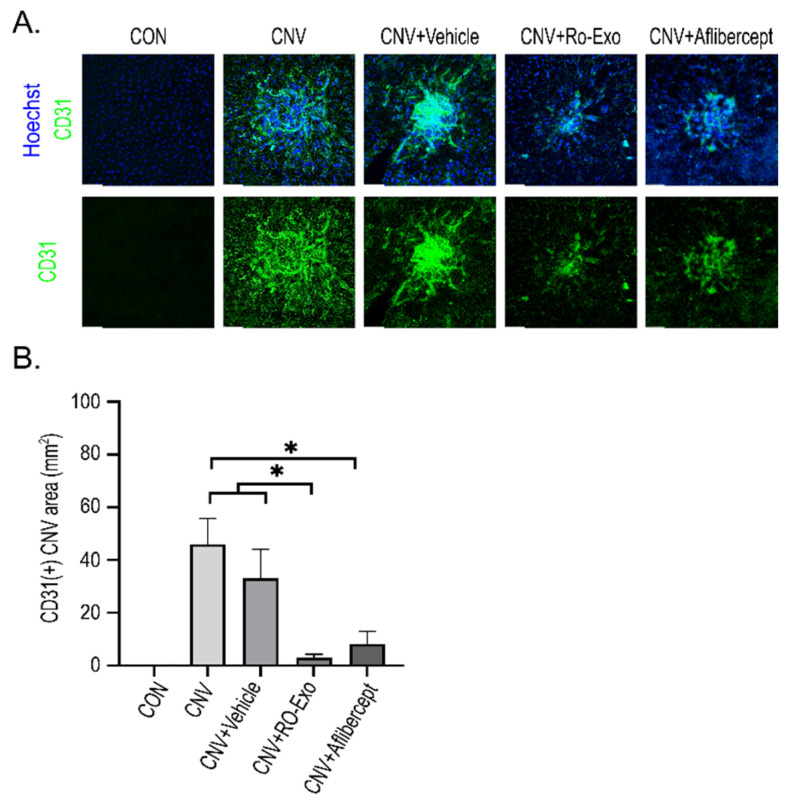
Anti-angiogenic effect of RO-Exo assessed by CD31 immunostaining. (**A**) Representative confocal images of RPE–choroid flat mounts stained for CD31 (green) and Hoechst (blue) at day 12 post-LP. Scale bar = 50 µm. (**B**) Quantitative analysis of CD31-positive area (mm^2^). Data are presented as bar graphs showing mean ± standard error of the mean (S.E.M.). Statistical significance was denoted as *p* < 0.05 (*) using the Mann–Whitney U test.

To evaluate the effect of RO-Exo on CNV, we performed CD31 immunostaining on RPE–choroid flat mounts. CD31-positive vascular area was significantly increased in both CNV and CNV + vehicle groups compared to the control group (*p* < 0.05), confirming the induction of abnormal neovascularization. RO-Exo treatment significantly reduced the CD31-positive area compared to both the CNV groups (w/wo vehicle) (*p* < 0.05), and no significant difference was observed between the RO-Exo and aflibercept groups (Figure 5A,B). These findings suggest that RO-Exo effectively suppresses pathologic neovascularization in the CNV model, comparable to the effect of aflibercept.

### 2.6. Restoration of RPE Monolayer Structure and Morphology by RO-Exo

**Figure 6:**
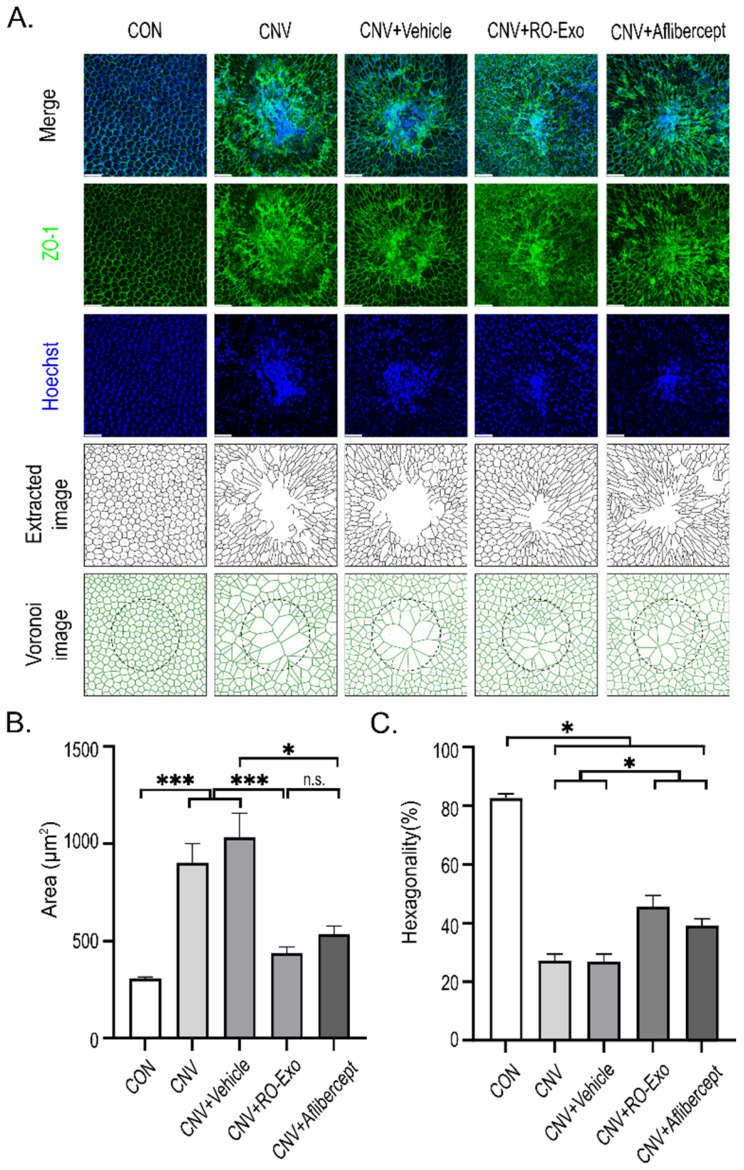
RO-Exo promotes morphological restoration of RPE cells. (**A**) ZO-1 (green) and Hoechst (blue) staining of RPE–choroid flat mounts with corresponding extracted and Voronoi images showing RPE cell morphology across groups. Scale bar = 50 µm. (**B**) Quantification of RPE cell area (µm^2^). n.s. indicate not significant. (**C**) Quantification of RPE cell hexagonality (%). Higher values indicate greater morphological regularity. Data are presented as bar graphs showing mean ± standard error of the mean (S.E.M.). Statistical significance was denoted as *p* < 0.05 (*), and *p* < 0.001 (***) using the *t*-test.

To evaluate the structural integrity and morphological changes in the RPE in a laser-induced CNV model, ZO-1 immunostaining was performed on RPE flat mounts to assess the preservation of tight junctions and monolayer architecture. In control (CON) eyes, confocal imaging revealed that RPE cells displayed a densely packed, hexagonal morphology with clearly demarcated ZO-1-positive boundaries. In contrast, CNV-induced groups (w/wo vehicle) exhibited substantial structural disruption, including irregular cell shapes, increased cell size heterogeneity, and impaired cell–cell contacts around the CNV lesion. In the RO-Exo–treated group, epithelial alignment was improved and ZO-1 signals were more continuous, indicating partial restoration of tight junctions and monolayer integrity. Although aflibercept-treated eyes also exhibited partial recovery of ZO-1 signals, monolayer organization remained poorly defined in peripheral regions surrounding the lesion, suggesting a less robust structural restoration compared to RO-Exo (Figure 6A).

Quantitative analysis revealed that CNV induction significantly increased RPE cell area compared to the CON group (*** *p* < 0.001), indicating cellular hypertrophy. RO-Exo treatment markedly reduced the enlarged cell area, showing significant differences compared to both CNV and CNV + Vehicle groups (*** *p* < 0.001). In contrast, aflibercept significantly reduced cell area relative to CNV + Vehicle (* *p* < 0.05), but not relative to the CNV group, suggesting a partial effect (Figure 6B).

Furthermore, the percentage of hexagonal cells—a key morphological index of RPE monolayer integrity—was significantly reduced in all CNV-induced groups compared to control (* *p* < 0.05 vs. CON), reflecting disruption of the monolayer architecture. RO-Exo treatment significantly increased the proportion of hexagonal cells relative to the CNV group (* *p* < 0.05), indicating partial restoration of typical RPE morphology. Aflibercept also significantly improved hexagonality compared to the CNV group, although to a lesser extent than RO-Exo (Figure 6C).

### 2.7. Suppression of Epithelial–Mesenchymal Transition in RPE Cells by RO-Exo

**Figure 7:**
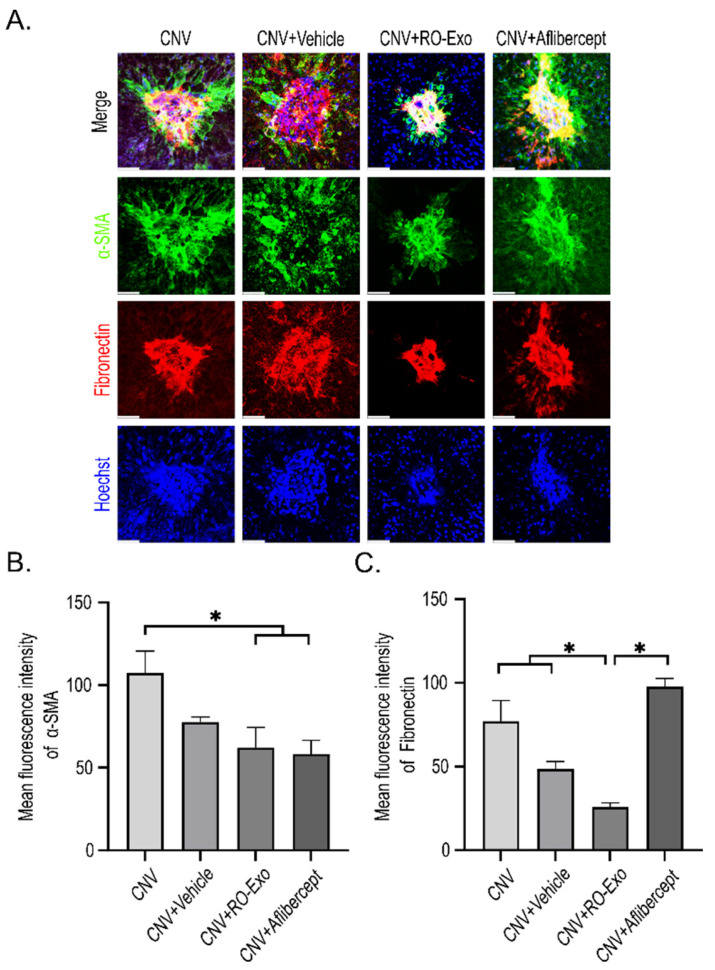
RO-Exo inhibits EMT in the RPE. (**A**) Confocal images of α-SMA (green) and fibronectin (red) staining in RPE–choroid flat mounts at day 12 post-LP. Scale bar = 50 µm. (**B**,**C**) Quantification of α-SMA-positive (**B**) and fibronectin-positive (**C**) areas normalized to total RPE area. Data are presented as bar graphs showing mean ± standard error of the mean (S.E.M.). Statistical significance was denoted as *p* < 0.05 (*) using the Mann–Whitney U test.

To evaluate epithelial–mesenchymal transition (EMT) responses in RPE following CNV induction, immunofluorescent staining for α-smooth muscle actin (α-SMA) and fibronectin was performed (Figure 7A). Both markers were markedly upregulated in the CNV groups (w/wo vehicle) indicating EMT activation.

RO-Exo treatment significantly reduced the expression of both α-SMA and fibronectin (*p* < 0.05), whereas aflibercept partially suppressed α-SMA but not fibronectin, which remained significantly higher compared to the RO-Exo group (Figure 7B,C).

These findings suggest that RO-Exo effectively attenuates EMT-related changes and contributes to the preservation of RPE structure.

### 2.8. Inhibition of MAPK Pathway RO-Exo

**Figure 8:**
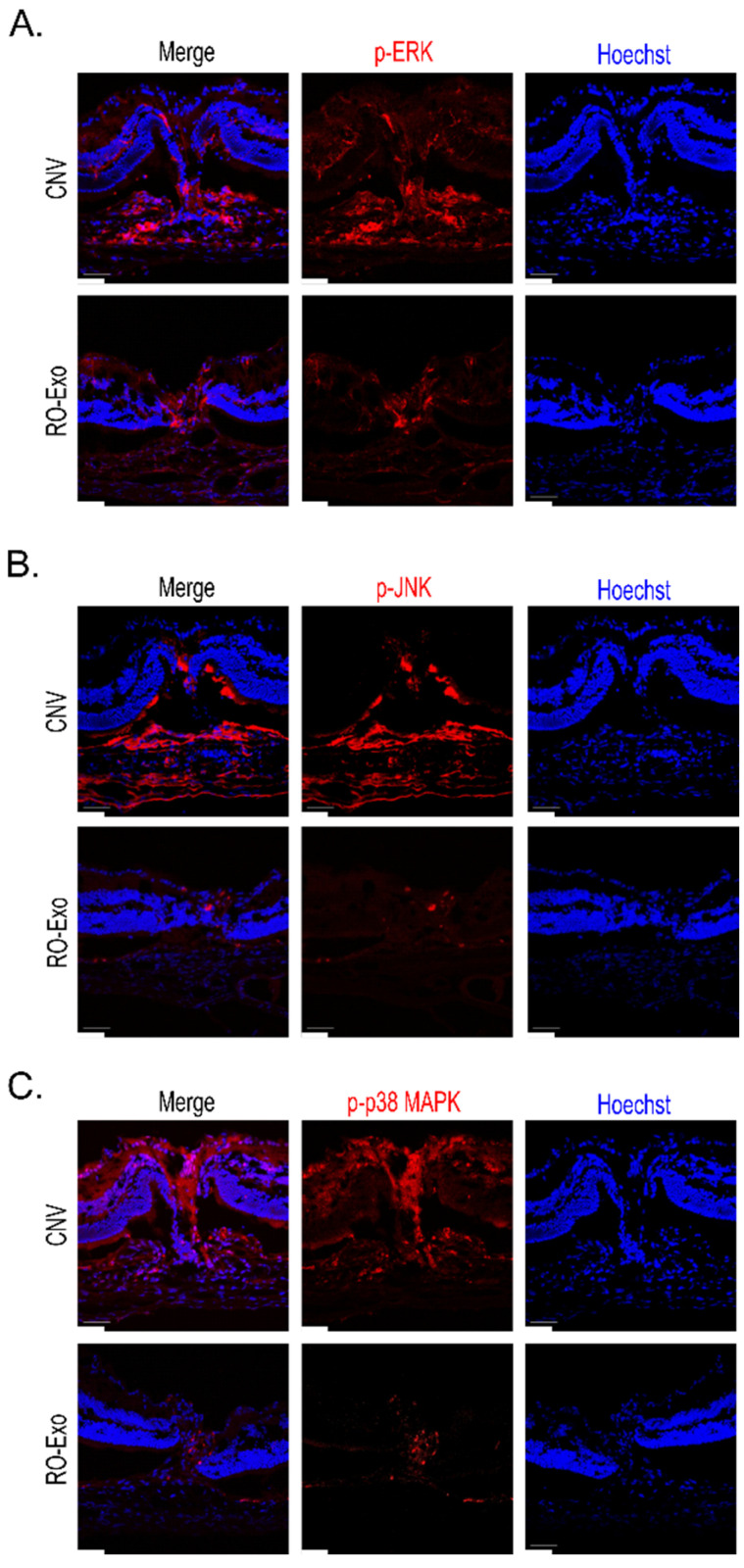
RO-Exo reduces phosphorylation of MAPK pathway proteins in CNV retina. (**A**–**C**) Representative confocal images of phosphorylated ERK (**A**), JNK (**B**), and p38 MAPK (**C**) in retinal sections at day 12 post-laser photocoagulation (LP). p-ERK, p-JNK, and p-p38 MAPK (red) were highly expressed in CNV lesions, whereas RO-Exo treatment reduced their expression levels. Hoechst (blue) was used for nuclear staining. Scale bar = 50 µm.

To confirm the mitogen-activated protein kinase (MAPK) pathway, immunohistochemistry was conducted in CNV retina at day 12 after laser photocoagulation. Antibodies specific for the phosphorylated forms of ERK, JNK and p38 were stained in CNV lesions and the positive signals on p-ERK, p-JNK and p-p38 were present in CNV lesions. The positive phosphorylation signals were decreased in RO-Exo-treated CNV retina at day 7 after IVT (Figure 8).

These results demonstrated that RO-Exo inhibited the activation of the MAPK pathway in CNV retina.

In neovascular (wet) age-related macular degeneration (AMD), damage to the retinal pigment epithelium (RPE) and the formation of fibrotic scars resulting from pathological neovascularization are critical therapeutic targets. These processes contribute to photoreceptor degeneration and progressive vision loss, underscoring the need for interventions that preserve retinal integrity and function. While anti-VEGF therapies are standard, they often fall short in addressing subretinal fibrosis and RPE degeneration, underscoring the need for multi-targeted treatments [5,26,27].

Our previous work characterized exosomes derived from retinal organoids (RO-Exo) and provided foundational evidence supporting their potential application in the treatment of retinal diseases. This analysis revealed the presence of multiple microRNAs (miRNAs) within RO-Exo, which are implicated in the regulation of diverse cellular processes and components critical for maintaining retinal structure and function [25]. Building upon these findings in our previous study, the present study demonstrates that exosomes derived from human induced pluripotent stem cell (iPSC)–retinal organoids exert therapeutic effects in a choroidal neovascularization (CNV) model.

Following intravitreal injection, RO-Exo were efficiently internalized by both retinal and retinal pigment epithelium (RPE) cells, indicating strong tissue tropism and promising delivery capabilities. And we confirmed the overexpression of hypoxia-inducible factor 1-alpha (HIF-1α) and vascular endothelial growth factor A (VEGFA)—key mediators of retinal neovascularization—and demonstrated the modulatory effects of RO-Exo on these pathological processes. RO-Exo inhibited the neovascular area and the scar formation in the subretinal space of the CNV lesion, which we confirmed by immunofluorescence staining using antibody markers.

In CNV, RPE degeneration and cellular transformation associated with CNV have been extensively documented. Drusen-induced RPE dysfunction is a well-established precursor to CNV formation and represents a hallmark of neovascular age-related macular degeneration (AMD). The hypoxia-induced pro-angiogenic pathway involving HIF-1α and VEGFA in the RPE–choroid complex is known to be upregulated during the progression of CNV and is associated with increased CNV-associated vascular leakage [28,29]. These findings are consistent with previous studies demonstrating that exosomes derived from neural or ocular sources can modulate angiogenesis by acting on endothelial cells [18,20,30].

Importantly, our findings demonstrate that RO-Exo treatment significantly restored the structural integrity and characteristic morphology of the RPE monolayer. CNV induction led to detrimental changes in RPE cells, including increased cell area and a significant reduction in the percentage of hexagonal cells, indicative of monolayer disruption and cellular hypertrophy. The remarkable ability of RO-Exo to ameliorate these pathological alterations, particularly in restoring RPE cell size, spatial arrangement, and, critically, hexagonal patterning, underscores their potential to directly support RPE health. The hexagonal shape of RPE cells is crucial for efficient nutrient and waste exchange, barrier function, and overall retinal homeostasis; thus, its preservation or restoration is a key indicator of therapeutic success and a critical factor for long-term retinal health [31,32,33].

Despite these encouraging results, several limitations must be considered. First, the RO-Exo used in this study was derived from retinal organoids differentiated at a specific developmental stage (D55–D65), and the molecular composition and therapeutic efficacy of exosomes are likely to vary depending on the degree of organoid maturation [23,34]. In addition, as these exosomes were produced from human iPSC-derived retinal organoids and administered to a mouse model, the xenogeneic nature of the system introduces potential immunological limitations. No assessment of immune responses was conducted in this study, and the possibility of cross-species immunogenicity cannot be excluded [35,36].

Second, the laser-induced CNV model used in this study represents an acute injury model and does not fully recapitulate the chronic and multifactorial pathophysiology of human neovascular AMD. Therefore, to more accurately evaluate the translational potential of RO-Exo, long-term studies using chronic disease models or large animal models are required to assess their therapeutic efficacy and safety [37,38].

In summary, RO-Exo also reduced CNV-associated vascular leakage and downregulated HIF-1α and VEGFA expression, indicating potent anti-angiogenic activity. The reduction in CD31-positive vascular area further supports RO-Exo’s ability to regress CNV lesions through both VEGF-dependent and independent mechanisms. Furthermore, an increased activation of the mitogen-activated protein kinase (MAPK) pathway was observed in the laser-induced CNV model. This activation was confirmed by immunohistochemistry against phospho-JNK, p-38 and phospho-ERK in the CNV lesion. RO-Exo inhibited MAPK activation and CNV size in the CNV model.

Exosomes derived from retinal organoids have a potential therapeutic effect in retinal diseases by delivering biomolecules, microRNAs and proteins. Here we showed their potential therapeutic efficacy through anti-angiogenic and a repairing effect on damaged RPE in laser-induced choroidal neovascularization.

These findings suggest that RO-Exo may overcome the major limitations of existing anti-VEGF monotherapy by simultaneously modulating multiple pathological pathways in neovascular AMD.

## 3. Materials and Methods

### 3.1. Retinal Organoid and Exosome Preparation

#### 3.1.1. Human Induced Pluripotent Stem Cells Culture and Retinal Organoid Differentiation

Human induced pluripotent stem cells (hiPSCs; ATCC DYR0100; American Type Culture Collection, Manassas, VA, USA) were maintained on vitronectin-coated plates in Essential 8 medium (Gibco, Thermo Fisher Scientific, Waltham, MA, USA). Retinal organoids (RO) were generated as previously described [39]. Embryoid bodies were differentiated in Neural Induction Medium, transferred to Matrigel-coated dishes in Neuroretina Medium, and manually isolated as optic vesicle-like structures between days 25–30. These were further cultured in Retinal Organoid Medium containing exosome-depleted fetal bovine serum (FBS). Conditioned medium was collected on days 55–65 and stored at −80 °C.

#### 3.1.2. Exosome Isolation

Conditioned medium was sequentially centrifuged at 300× *g* for 10 min, 2000× *g* for 10 min, and 10,000× *g* for 30 min. Exosomes were isolated using the miRCURY Exosome Isolation Kit (Qiagen, Hilden, Germany), and pellets were resuspended in 100 μL of a 1:1 mixture of suspension buffer and phosphate-buffered saline (PBS).

#### 3.1.3. Nanoparticle Tracking Analysis and Western Blotting

Exosome size and concentration were analyzed using a NanoSight NS300 (Malvern Panalytical, Malvern, UK). Western blotting was conducted on RO and RO-exosome lysates using antibodies against CD9, CD63, TSG101, and calnexin (Cell Signaling Technology, Danvers, MA, USA), and β-actin (Santa Cruz Biotechnology, Dallas, TX, USA).

#### 3.1.4. Intravitreal Delivery of Labeled Exosomes

Exosomes (1 × 10^9^ particles) were labeled with 1μM Calcein-AM (Life Technologies, Carlsbad, CA, USA) at 37 °C for 30 min, washed with PBS, and re-isolated using the miRCURY kit. Labeled exosomes (1 μL) were injected intravitreally into wild-type (WT) C57BL/6 J mice. Retinas were collected 3 h post-injection and imaged using confocal microscopy (TCS SP8; Leica Microsystems, Wetzlar, Germany).

### 3.2. Experimental Design and Treatment for In Vivo Study

#### 3.2.1. Animals

All animal procedures followed the ARVO statement for the Use of Animals and were approved by the Soonchunhyang University IACUC (SCHBCA 2020-05). Eight-week-old male C57BL/6 J mice (DBL Inc., Eumseong, Republic of Korea) were used. All animals were kept in a specific pathogen-free (SPF) animal facility with free access to food and water and maintained under 12/12 h light/dark cycles. Prior to all examinations, anesthesia was induced via intraperitoneal injection of a mixture containing 30 mg/kg Alfaxan (Zoetis, Parsippany-Troy Hills, NJ, USA) and 10 mg/kg Rompun (Bayer Healthcare, Leverkusen, Germany), and pupil dilation was performed with a mixture of 0.5% (*w*/*v*) tropicamide and 0.5% (*w*/*v*) phenylephrine (Hanmi Pharm, Seoul, Republic of Korea).

#### 3.2.2. Laser Photocoagulation and Intravitreal Injection

Laser photocoagulation (532 nm, 200 μm spot size, 120 mW, 20 ms, 5 spots per eye) was applied using a PASCAL system (SL-PA04; Topcon Medical Laser Systems, Livermore, CA, USA). Successful Bruch’s membrane rupture was confirmed by bubble formation. Five days after laser photocoagulation (post-LP), mice were administered intravitreal injections of RO-Exo (1 × 10^9^ particles/μL), aflibercept (Regeneron Pharmaceuticals, Tarrytown, NY, USA), or PBS vehicle using a 34 G NanoFil syringe (World Precision Instruments, Sarasota, FL, USA) via a 33 G pre-puncture.

### 3.3. Fundus Fluorescein Angiography

Fundus fluorescein angiography (FFA) was performed on days 5 and 12 post-laser photocoagulation (LP). Mice were anesthetized, pupils dilated, and 2% fluorescein sodium (Fluorescite; Akorn, Lake Forest, IL, USA) was injected intraperitoneally. Fluorescent images were captured using the Heidelberg Retina Angiograph 2 system; Heidelberg Eye Explorer software (Rev. 0.98.0.1) Heidelberg Engineering, Heidelberg, Germany) over a 5 min period. The clearest late-phase image per eye was selected. Lesion area and intensity were quantified in ImageJ (v1.54f; NIH, Bethesda, MD, USA) by manually delineating a region of interest (ROI) and applying the “Set Scale” function based on the embedded scale bar to convert pixel values into micrometers.

### 3.4. Tissue Analysis by Immunofluorescence and Western Blotting

#### 3.4.1. Immunofluorescence

Mice were perfused with 4% paraformaldehyde (PFA) in phosphate buffer (PB; Biosesang, Seongnam, Republic of Korea). Eyes were fixed, cryoprotected in 30% sucrose (Duksan, Ansan, Republic of Korea), embedded in OCT compound (Leica Biosystems, Buffalo Grove, IL, USA), and sectioned at 10 μm. Tissues were washed in PBS, permeabilized with 0.1% Triton X-100 (Sigma-Aldrich, St. Louis, MO, USA) for 30 min, and blocked in 5% normal donkey serum (Jackson ImmunoResearch, West Grove, PA, USA) for 1 h. Tissues were incubated overnight at 4 °C with the following primary antibodies: ZO-1 (Invitrogen, Carlsbad, CA, USA), CD31 (BD Biosciences, San Jose, CA, USA), α-SMA (Sigma-Aldrich, St. Louis, MO, USA), fibronectin (Abcam, Cambridge, UK), and p-ERK, p-JNK, and p-p38 MAPK (Cell Signaling Technology, Danvers, MA, USA). Following PBS washing, Alexa Fluor-conjugated secondary antibodies (488 or 568; Invitrogen, Carlsbad, CA, USA) were used. Nuclei were counterstained with Hoechst 33342 (Invitrogen, Carlsbad, CA, USA) and tissues were mounted with a fluorescence mounting medium (Dako, Santa Clara, CA, USA). Confocal images were acquired using a Leica TCS SP8 microscope (Leica Microsystems, Wetzlar, Germany) under consistent imaging settings. Fluorescent signal intensity and lesion area were quantified using ImageJ software (National Institutes of Health, Bethesda, MD, USA). RPE morphology stained with ZO-1 was assessed using Voronoi diagrams generated in Rhinoceros software (v8.0; Robert McNeel & Associates, Seattle, WA, USA) and vectorized using Inkscape (v1.4; Inkscape Project, Boston, MA, USA). Cell area was measured using ImageJ.

#### 3.4.2. RPE Hexagonality

Hexagonality (%) was defined as the percentage of cells with a form factor value (4π × [area/perimeter^2^]) greater than 0.85 [40]. This approach follows previously established methods for assessing cellular polygonal regularity and epithelial integrity. For each sample, three circular regions (200 μm in diameter) centered on the CNV lesion were analyzed. Cells at the image margins or with ambiguous boundaries were excluded from the analysis.

### 3.5. Western Blot Analysis of RPE–Choroid Complex

RPE-choroid tissues were lysed in Radioimmunoprecipitation assay buffer (RIPA) with inhibitors (GenDEPOT, Barker, TX, USA), sonicated, and quantified using Bicinchoninic acid assay (BCA; Thermo Fisher Scientific, Waltham, MA, USA). Proteins were separated by Sodium dodecyl sulfate–polyacrylamide gel electrophoresis (SDS-PAGE), transferred to Polyvinylidene difluoride membranes (PVDF; Millipore, Burlington, MA, USA), and probed with antibodies against HIF1-α, VEGFA (Abcam, Cambridge, UK), and β-actin (Santa Cruz Biotechnology, Dallas, TX, USA). HRP-conjugated secondary antibodies (GenDEPOT, Barker, TX, USA) and chemiluminescent substrate (EzWestLumi plus; ATTO, Tokyo, Japan) were used for detection.

### 3.6. Statistical Analysis

All experiments were repeated ≥3 times. Data were analyzed using one-way analysis of variance (ANOVA) or paired *t*-test (SPSS v22.0; IBM Corp., Armonk, NY, USA). Kruskal–Wallis tests followed by Mann–Whitney U were used for Western Blot and immunofluorescence. Data are shown as mean ± standard error of the mean (S.E.M.). Significance was indicated as * *p* < 0.05, ** *p* < 0.01, *** *p* < 0.001.

## 4. Conclusions

In conclusion, this study demonstrated the multi-modal therapeutic efficacy of human iPSC-retinal organoid-derived exosomes (RO-Exo) in a laser-induced choroidal neovascularization (CNV) model. We showed that RO-Exo effectively attenuated key pathological processes by suppressing neovascularization and inhibiting the MAPK pathway, similar to the anti-VEGF agent aflibercept. More significantly, RO-Exo restored the structural integrity and characteristic morphology of the RPE monolayer and suppressed the detrimental Epithelial–Mesenchymal Transition (EMT) response, indicating a unique tissue-repairing capacity beyond anti-VEGF monotherapy. Taken together, our findings suggest that RO-Exo could serve as a potent, multi-targeted platform for the development of therapeutics against neovascular AMD.

## Data Availability

The original contributions presented in this study are included in the article. Further inquiries can be directed to the corresponding author.
